# Serum Malondialdehyde Concentration and Glutathione Peroxidase Activity in a Longitudinal Study of Gestational Diabetes

**DOI:** 10.1371/journal.pone.0155353

**Published:** 2016-05-26

**Authors:** Luis Arribas, Inmaculada Almansa, María Miranda, María Muriach, Francisco J. Romero, Vincent M. Villar

**Affiliations:** 1 Unidad de Diabetes, Servicio de Medicina Interna, Hospital de La Plana, Vila-real, Spain; 2 Dept. Ciencias Biomédicas, Universidad CEU Cardenal Herrera, Valencia, Spain; 3 Dept. Medicina, Universidad Jaume I, Castelló, Valencia, Spain; 4 Facultad de Medicina, Universidad Católica de Valencia “San Vicente Martir”, Valencia, Spain; Kermanshah University of Medical Sciences, ISLAMIC REPUBLIC OF IRAN

## Abstract

**Aims:**

The main goal of this study was to evaluate the presence of oxidative damage and to quantify its level in gestational diabetes.

**Methods:**

Thirty-six healthy women and thirty-six women with gestational diabetes were studied in the three trimesters of pregnancy regarding their levels of oxidative stress markers. These women were diagnosed with diabetes in the second trimester of pregnancy. Blood glucose levels after 100g glucose tolerance test were higher than 190, 165 or 145 mg/dl, 1, 2 or 3 hours after glucose intake.

**Results:**

The group of women with gestational diabetes had higher serum malondialdehyde levels, with significant differences between groups in the first and second trimester. The mean values of serum glutathione peroxidase activity in the diabetic women were significantly lower in the first trimester. In the group of women with gestational diabetes there was a negative linear correlation between serum malondialdehyde concentration and glutathione peroxidase activity in the second and third trimester.

**Conclusions:**

In this observational and longitudinal study in pregnant women, the alterations attributable to oxidative stress were present before the biochemical detection of the HbA1c increase. Usual recommendations once GD is detected (adequate metabolic control, as well as any other normally proposed to these patients) lowered the concentration of malondialdehyde at the end of pregnancy to the same levels of the healthy controls. Serum glutathione peroxidase activity in women with gestational diabetes increased during the gestational period.

## Introduction

Although oxidants and antioxidants have been studied extensively in Type II Diabetes and its complications, only limited data are available for Gestational Diabetes (GD), a disease of similar pathophysiology.

In patients with diseases that complicate pregnancy, several elevated markers of oxidative stress have been found [1–3]. Chen and collaborators [4] detected in a prospective study of 408 healthy pregnant women that the glutathione peroxidase (GPx) activity in erythrocytes of the 16^th^ and 30^th^ week of pregnancy, positively correlated with insulin-resistance markers, suggesting a relationship between resistance to insulin and antioxidant defence in a non-diabetic pregnant woman, though it is still unclear which type of relationship exists between diabetes and oxidative stress [5–10].

Several *in vitro* studies have used placental tissue to measure oxidative stress after the GD diagnosis was performed [11–14]. Coughlan et al [11] found that the 8-isoprostane release by the placenta increased 2-fold in women with GD with respect to the healthy group. The same research group [12] showed that the placenta of women with GD exposed to Superoxide-Generating Systems (xanthine and xanthine oxidase) have a reduced response to oxidative stress. Similarly, Lappas and coworkers [13] showed that placenta, fat and muscle tissues released, upon incubation, more 8-isoprostane in women with GD than in healthy ones.

There are some evidences which suggest that oxidative stress in GD is accompanied by changes in the antioxidant capacity [15–19]. Kamath et al. collected maternal erythrocytes immediately after giving birth and found that the proteolytic activity, which is an indicator of protein oxidation, was increased in women with GD, and that malondialdehyde (MDA) concentration in erythrocytes was higher in GD but not significantly different with respect to the healthy women [20].

Peuchant et al. showed how the maternal plasmatic MDA concentration was increased and the erythrocytic GPx activity was diminished in GD as well as in type I diabetes, between weeks 26 and 32 of gestation [17]. Nevertheless, there was no difference in erythrocytic and plasmatic GPx and catalase activities or thiobarbituric acid reactive substances (TBARS) concentration during the third trimester. Toescu and co-workers [15] reported a longitudinal study by trimesters, and found that the plasmatic and lipid hydroperoxides were higher in each trimester for women with type I, type II and GD, compared with the pregnant control group, but the antioxidant capacity was significantly decreased. Epidemiological data also support the relationship between the plasmatic decrease of vitamin C concentration and the increased risk of showing symptoms of GD [21].

The level of maternal glycemic control has been correlated with several markers of oxidative stress in GD: erythrocytic MDA content with glycosylated haemoglobin HbA1c [17], and 8-isoprostane in placenta with maternal glycemia 2 hours after the oral glucose tolerance test (OGTT) [11].

The decrease of antioxidant defence reported in studies of women with GD could indicate the consumption effect exerted by the already existing oxidative stress.^17^ It is not clear if the level of glycemic control in GD is associated with the severity of oxidative stress as it occurs in type II diabetes [22].

The main purpose of the present study was to evaluate if there was oxidative damage and, if so, to measure its extent in gestational diabetes.

## Methods

### Subjects

We screened 808 pregnant women during the first trimester of pregnancy. All procedures performed were in accordance with the ethical standards of the institutional and/or national research committee and with the 1964 Helsinki declaration and its later amendments or comparable ethical standards. Study was approved by the Institutional Review Board of the Universidad CEU Cardenal Herrera on May 2001. Only 16% (126 women) completed the study during the whole pregnancy. Women with a history of diabetes before pregnancy or with pre-existing hypertension, urinary infections, renal disease, autoimmune disorders, hypothyroidism or any other type of disorder, and smokers were excluded from this work, so only 8.9% (72 women) were suitable for the present study, none of the women had an acute disease or clinical complication during pregnancy.

We screened for GD in all nondiabetic pregnant women using a two-step standard protocol. At 24–28 weeks, all pregnant women without previously diagnosed diabetes were offered screening for GD with a 1-h 50 g glucose challenge test during a routine prenatal visit. A value of 140 mg/dL (7.8 mmol/L) identified patients who undergo a 3 h 100 g diagnostic OGTT. GD was diagnosed when two or more plasma glucose values during the diagnostic OGTT met or exceeded the criteria for a positive test, as recommended by the National Diabetes Data Group (plasma glucose thresholds: fasting 5.8mmol/L (105 mg/dL), 1-h 10.5 mmol/L (190 mg/dL), 2-h 9.1 mmol/L (165 mg/dL), 3-h 8.0 mmol/l (145 mg/dL)). All pregnant women and GD patients were advised to control caloric intake and GD patients additionally to monitor blood glucose levels [23].

Thirty six healthy pregnant women (age-matched healthy pregnant women served as controls) and thirty seven women with GD, diagnosed in the 2nd trimester were studied in the three trimesters of pregnancy regarding their levels of oxidative stress markers (blood samples were taken during weeks 12–14, 24–26 and 36–40 of pregnancy in fasting conditions). All of them gave written informed consent to participate in the study. All of them received the same polivitaminic treatment and the same dietary recommendations and performed the same physical exercise. There were no statistically significant differences in maternal age, parity and sex and in the body weight of the newborns (Table 1). Women with twin pregnancy were excluded from the study.

**Table 1:** Demographic characteristics of healthy pregnant and diabetes pregnant women (GD).

Variables			Mean ± SD	CI 95%		
				Lower limit	Upper limit	p value
Mother age (years)	GD		32.86 ± 4.26	31.42	34.30	ns
	Control		29.70 ± 4.64	28.05	31.34	
BMI (kg/m^2^)	GD		27.3 ± 9.9	24.2	31.5	ns
	Control		24.4 ± 3.9	23.1	26.1	
Weight gain (kg)	GD		10.0 ± 0.35	9.8	10.4	ns
	Control		9.7 ± 0.043	9.6	9.9	
Gestation length (days)	GD		275.03± 11.54	271.12	278.93	ns
	Control		278.85± 11.47	274.78	282.92	
Newborn weight (g)	GD		3178.61 ± 542.50	2995.05	3362.16	ns
	Control		3287.15 ± 517.38	3100.61	3473.69	
Newborn sex M/W	GD	19(52.8%)/17 (47.2%)				ns
	Control	21(58.4%)/15(41.6%)				
Delivery start (I/S)	GD	24 (66.6%)/12 (33.4%)				ns
	Control	26 (72.2%)/10 (27.8%)				
Delivery type (V/C)	GD	26 (72.2%)/10 (27.8%)				ns
	Control	27(75%)/9 (25%)				
Parity	GD	1.6	0.8	1	3	ns
	Control	1.8	0.7	1	3	

All women (36 women each group) belonged to the same race, all were Caucasian. (CI = confidence interval; ns = not significant; BMI = body mass index; M/W = male/women; I/S = induced/spontaneous; V/C = vaginal/caesarean). Values are given as mean ± standard deviation.

### Biochemical assays

Seric MDA concentration, a lipid peroxidation product, was determined by liquid chromatography according to a modification of the method of Richard et al. [24] as previously described [25]. Briefly, 0.1 ml of sample (or standard solutions prepared daily from 1,1,3,3-tetramethoxypropane and 0.75 ml of working solution (thiobarbituric acid 0.37% and perchloric acid 6.4%, 2:1, v/v) were mixed and heated to 95°C for 1 h. After cooling (10 min in ice-water bath), the flocculent precipitate was removed by centrifugation (3200 g, 10 min). The supernatant was neutralized and filtered (0.22 μm) before injection on an ODS 5-μm column (250×4.6mm). Themobile phase consisted in 50 mM phosphate buffer, pH 6.0: methanol (58:42, v/v). Isocratic separation was performed at 1.0 ml /min flow (HPLC System 325; Kontron, Germany) and detection at 532 nm (UV/Vis HPLC Detector 332; Kontron, Germany). Calibration curves were run daily. GPx activity in serum was assayed as reported by Lawrence et al. [26] towards hydrogen peroxide. The oxidation of NADPH was followed spectrophotometrically at 340 nm. The reaction mixture consisted of 240 mU/ml glutathione disulfide reductase, 1 mM GSH, 0.15 mM NADPH in 0.1 M potassium phosphate buffer, pH 7.0, containing 1 mM EDTA and 1 mM sodium azide; 50-μl samples were added to this mixture and allowed to equilibrate at 37°C for 3 min. Reaction was started by the addition of 1.5 mM hydrogen peroxide to adjust the final volume of the assay mixture to 1 ml.

### Statistical Analysis

All data are expressed as mean ± SD. The study of the relationship of the categorical variables was performed using the Pearson’s Chi-squared test, and the two-tailed Fisher’s exact test to improve statistical significance when the sample number is small as is this case (smaller that 50). Pearson’s linear regression analysis was used.

The Mann-Whitney U test for not normally distributed data was used, and Student’s t test for other continuous data. In the case of comparing one of the variables with more than two categories, with a continuous one, Kruskal-Wallis non-parametric test was used, comparing afterwards each pair with the Mann-Whitney test but applying the Tukey’s range test. Relative risks and 95 percent confidence intervals were calculated with SPSS software package version 12.0.1 (SPSS Inc. 2004, Chicago, IL, USA).

## Results

As it can be seen in Table 1 the two study groups were similar in the principal demographic and clinical characteristics. All women belonged to the same race, all were Caucasian.

**Fig 1:**
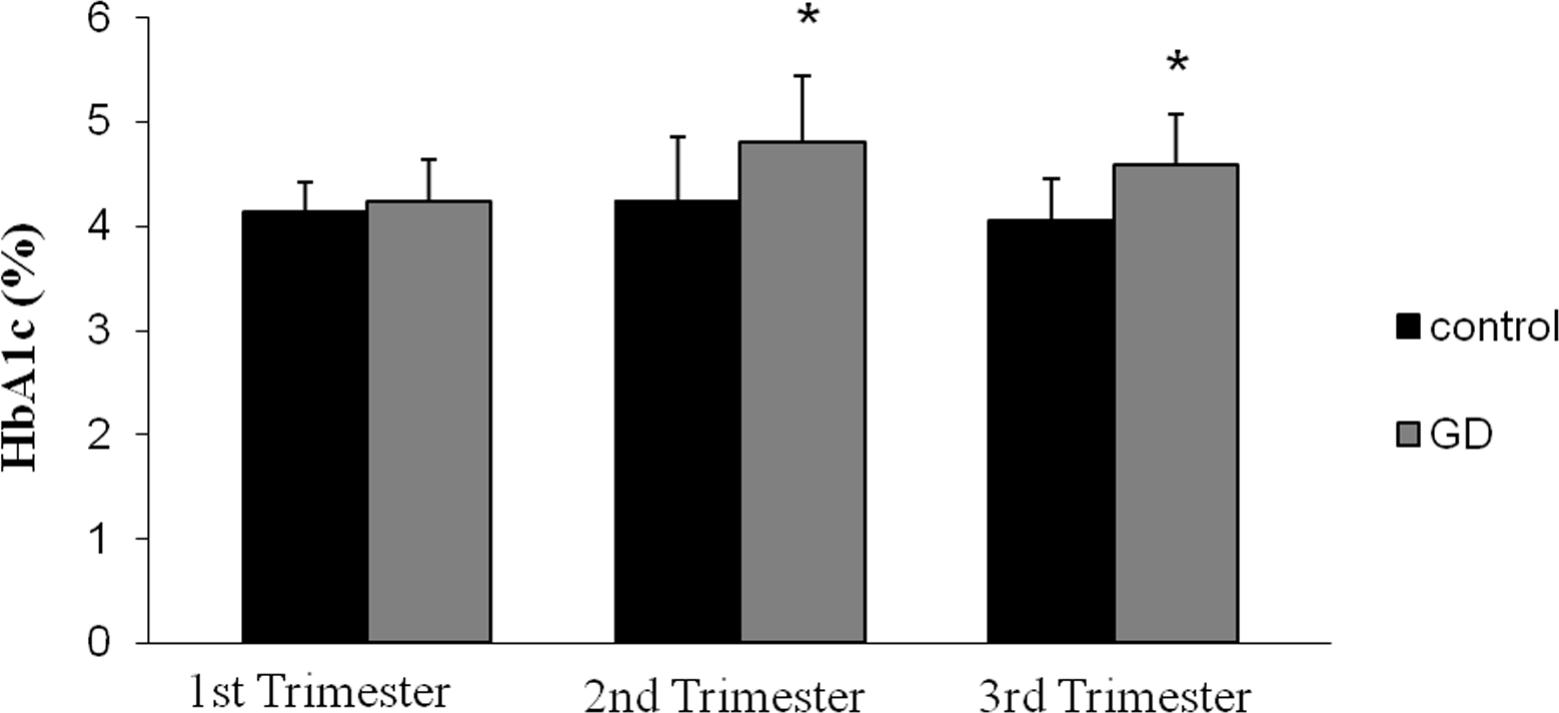
Percentage of Glycosylated Hemoglobin (HbA1c) in both groups of women (control and diabetic women) in the three trimesters of gestation. Data are expressed as mean ± SD (* p < 0.05 versus control).

The HbA1c levels were similar for both groups of women in the first trimester (before diabetes started), but significantly higher in the GD group in the second and third trimester than in healthy subjects (Fig 1).

**Fig 2:**
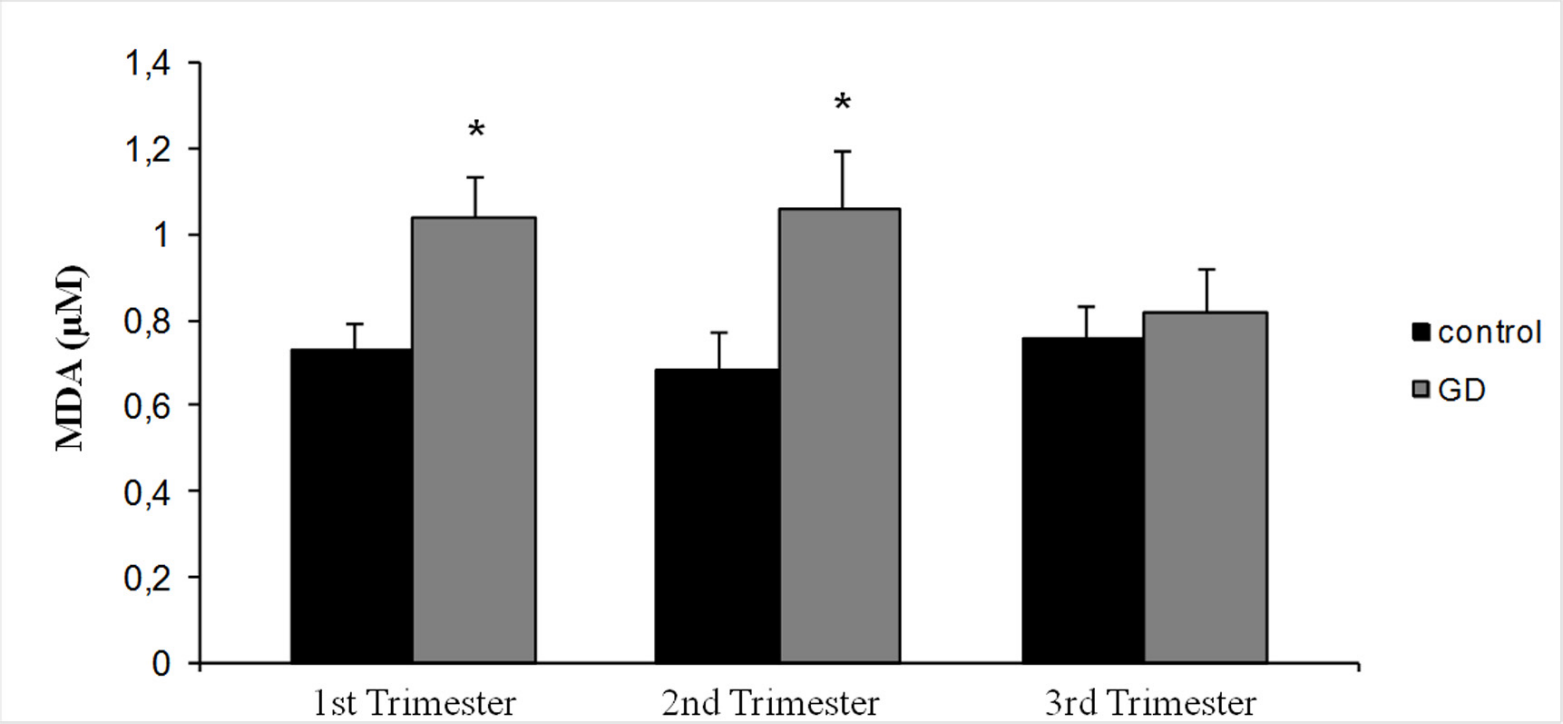
Mean values of MDA concentration in both groups of women (healthy and diabetic women) in the three trimesters of gestation. Data are expressed as mean ± SD (* p < 0.05 versus control).

**Fig 3:**
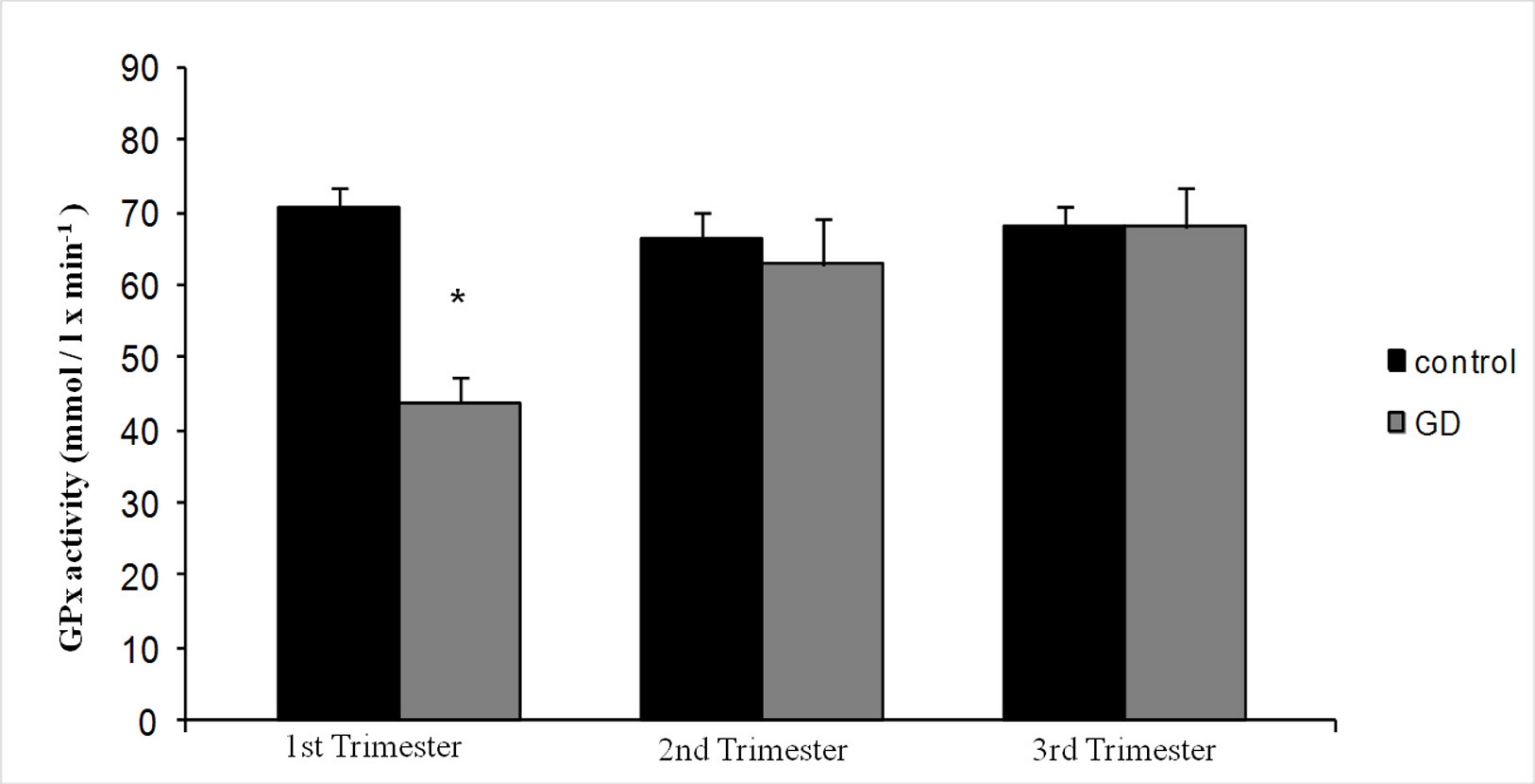
Mean values of Glutathione-Peroxidase (GPx) activity in both groups of women (healthy and diabetic women) in the three trimesters of gestation. Data are expressed as mean ± SD (*p < 0.05 versus control).

The mean values of MDA concentration were higher in the GD group during the whole period of gestation, with significant differences in the first and second trimesters with respect to the control group (Fig 2). Surprisingly, MDA levels of the GD group in the third trimester decreased to control values. The mean values of GPx activity for the diabetic women were always lower, with statistically significant differences in the first trimester respect to the control group (Fig 3). Moreover, GPx activity showed statistically significant differences in the GD group between the first and the following trimesters.

**Fig 4:**
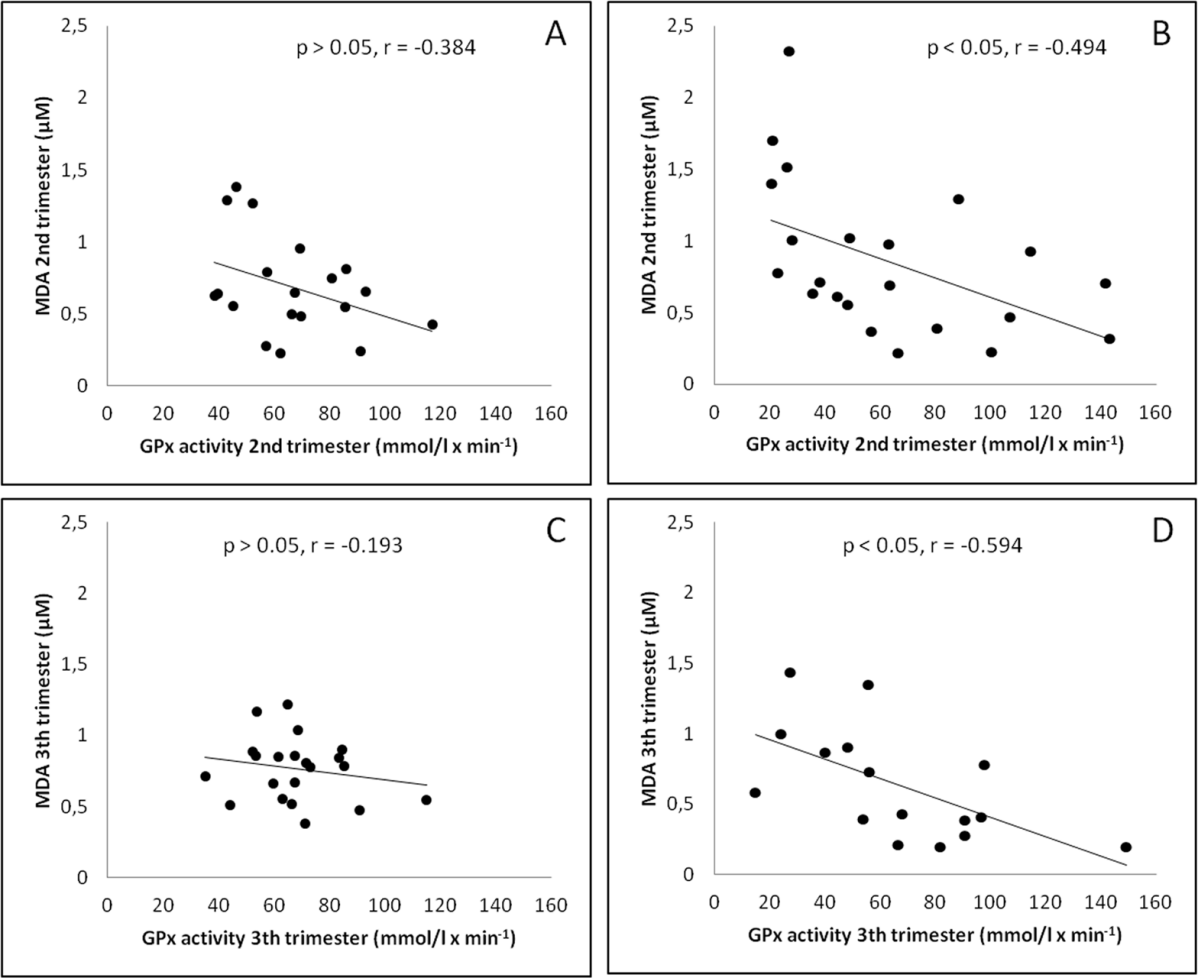
Correlation study through Pearson's linear regression analysis in the second and third trimester. (A) Correlation between MDA concentration and GPx activity in the second trimester at control group (p > 0.05, r = -0.384). (B) Correlation between MDA concentration and GPx activity in the second trimester at gestational diabetes group (p < 0.05, r = -0.494). (C) Correlation between MDA concentration and GPx activity in the third trimester at control group (p > 0.05, r = -0.193). (D) Correlation between MDA concentration and GPx activity in the third trimester at gestational diabetes group (p < 0.05, r = -0.594).

**Fig 5:**
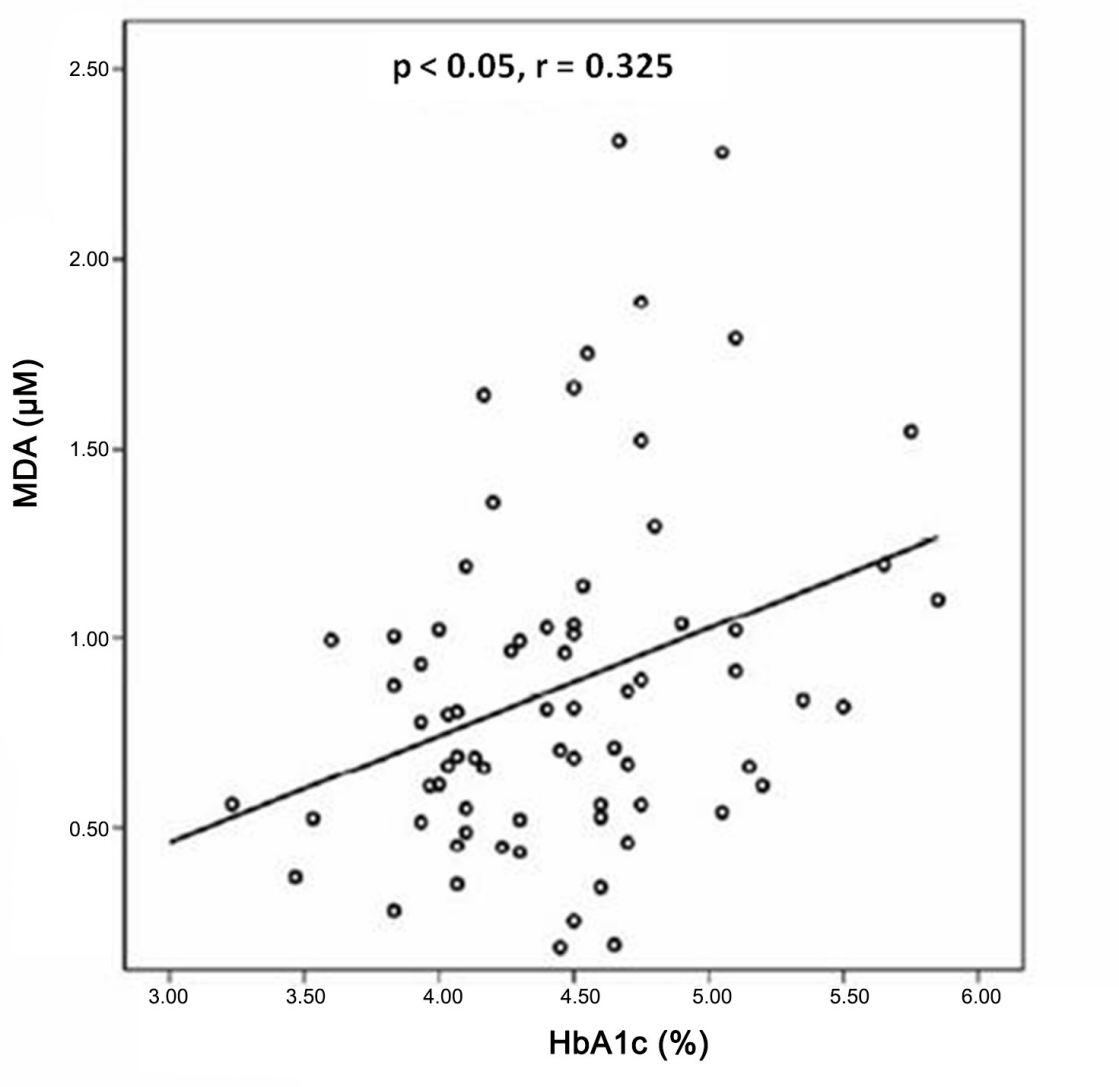
Correlation study through Pearson's linear regression analysis in all women between MDA concentration and percentage of HbA1c (p < 0.05, r = 0.325).

**Fig 6:**
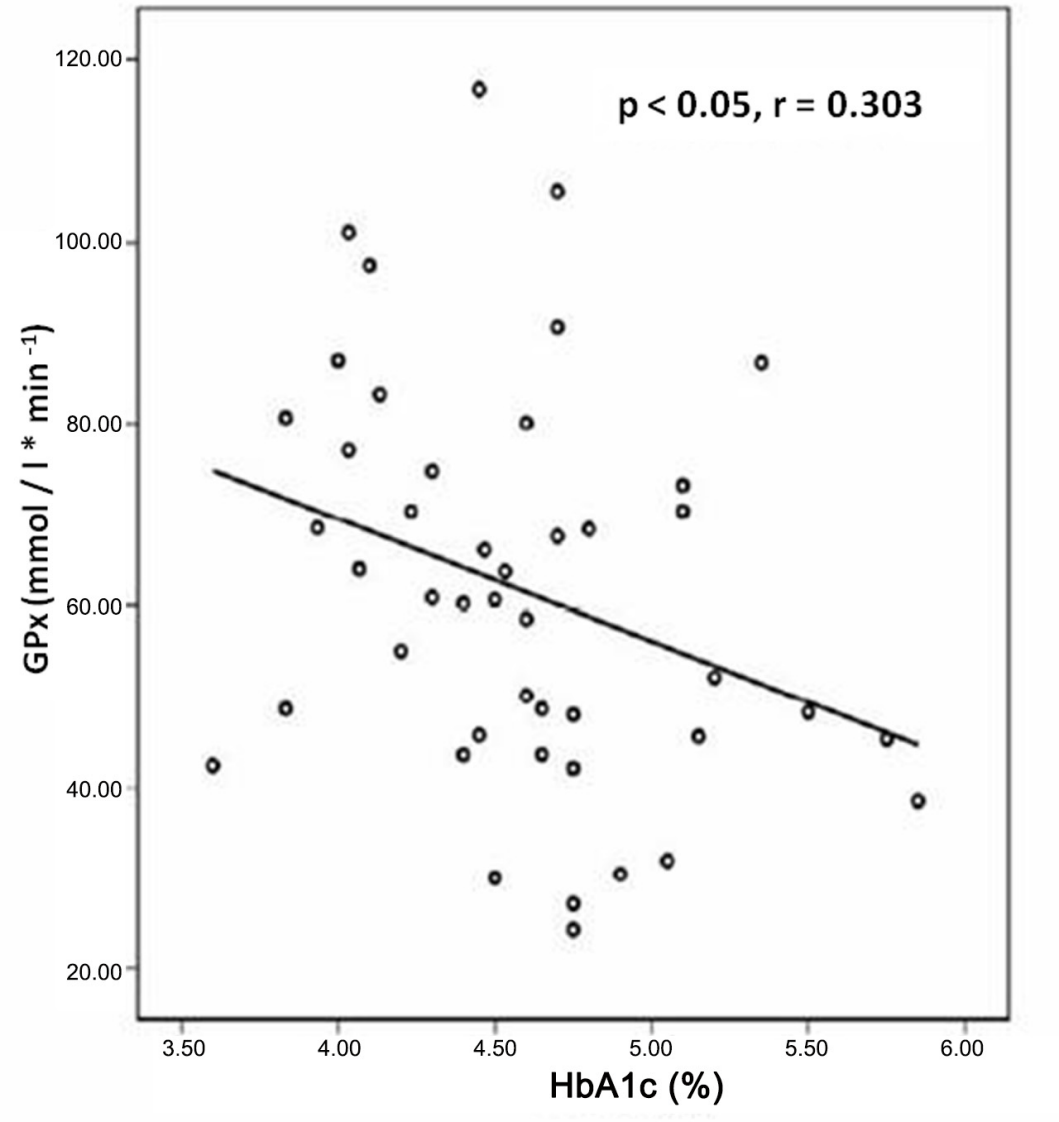
Correlation study through Pearson's linear regression analysis in all women between GPx activity and percentage of HbA1c (p < 0.05, r = 0.303).

The correlation study through Pearson's chi-square test showed that in the GD group there was a negative linear correlation between MDA levels and GPx activity in the second and third trimesters (p<0.05, r = -0.494, and p<0.05, r = -0.594) respectively (Fig 4B and 4D), whereas in the control group this negative linear correlation was not significant in the second and third trimesters (p>0.05, r = -0.384, and p>0.05, r = -0.193) (Fig 4A and 4C). When the GD and the control groups were evaluated together during the whole gestation, a direct correlation between MDA concentration and percentage of HbA1c was found (Fig 5) and an indirect one between GPx activity and HbA1c percentage were found (Fig 6).

The lipidic profiles of the patients were studied, and our results coincided with previously published data. Pregnancy progressively increases all the lipid components with no significant differences between groups of healthy and pregnant women with GD (Table 2) and there was no correlation between lipidic values and MDA concentration or GPx activity (data not shown).

**Table 2:** Biochemical variables of the two groups studied (36 women each group), healthy pregnant and diabetes pregnant women (GD).

Variables			Mean ± SD	CI 95%		
				Lower limit	Upper limit	p value
**Hb A1c (%)**	1^st^ trimester	Control	4.1±0.3	4.02	4.26	ns
		GD	4.2±0.4	3.98	4.49	
	2^nd^trimester	Control	4.2±0.6	4.04	4.46	p<0.001
		GD	4.8±0.6	4.59	5.02	
	3^rd^ trimester	Control	4.1±0.4	3.91	4.19	p<0.001
		GD	4.5±0.4	4.42	4.75	
**Total cholesterol (mg/dl)**	1^st^ trimester	Control	184.6±39.1	165.04	202.71	ns
		GD	172.5±23.4	162.34	182.22	
	2^nd^trimester	Control	226.3±38.1	209.21	245.76	ns
		GD	226.1±30.2	211.43	239.71	
	3^rd^ trimester	Control	264.2±40.6	274.95	283.33	ns
		GD	265.6±38.1	248.03	281.16	
**LDL cholesterol (mg/dl)**	1^st^ trimester	Control	92.8±21.8	82.37	102.67	ns
		GD	85.1±24.1	75.82	94.33	
	2^nd^trimester	Control	117.3±24.9	105.20	128.19	ns
		GD	114.8±32.9	100.61	128.51	
	3^rd^ trimester	Control	142.8±32.8	129.41	155.81	ns
		GD	144.5±41.2	126.69	163.36	
**HDL colesterol (mg/dl)**	1^st^ trimester	Control	68.3±17.4	62.01	78.43	ns
		GD	64.8±15.3	57.21	70.44	
	2^nd^trimester	Control	73.1±18.3	66.71	82.21	ns
		GD	70.1±15.5	65.99	79.32	
	3^rd^ trimester	Control	79.4±17.5	72.12	86.37	ns
		GD	76.4±16.3	69.60	83.67	
**Triglycerides (mg/dl)**	1^st^ trimester	Control	88.3±54.1	63.02	111.04	ns
		GD	83.9±32.7	67.36	94.51	
	2^nd^trimester	Control	152.1±47.6	131.82	170.32	ns
		GD	152.6±42.1	133.34	170.27	
	3^rd^ trimester	Control	206.1±70.5	171.49	220.15	ns
		GD	225.3±61.1	208.93	260.71	
**Fasting blood Glucose (mg/dl)**	1^st^ trimester	Control	83.1±6.1	80.99	85.24	ns
		GD	90.3±8.8	87.35	93.32	
	2^nd^trimester	Control	76.9±6.3	74.72	79.10	ns
		GD	87.2±9.9	83.91	90.65	
	3^rd^ trimester	Control	77.1±7.7	74.44	79.79	ns
		GD	83.9±12.2	79.77	88.06	

Values are given as mean ± standard deviation.

## Discussion

The main purpose of this study was to prove the existence of oxidative stress along pregnancy in GD by means of a longitudinal study. GD was approached by dividing it in three phases: the euglycemic one in the first trimester (before the GD diagnosis is performed), the starting hyperglycemia in the second trimester (in the moment of diagnosis, still without applying any treatment), and eventually the response to treatment in the third trimester. This study supports the hypothesis that there is an extra oxidative stress in GD, which could be counteracted by its treatment which then can significantly improve the outcome of pregnancy.

The percentage of HbA1c values in healthy pregnant women are lower than in the non-pregnant ones, but there has been some controversy about the time course of these values during pregnancy [27]. Some authors do not find statistically significant differences throughout the whole period of pregnancy [27], other authors find decreases [28, 29], and still others find increases during the third trimester [30, 31]. In our study both groups of pregnant women showed a small increase in HbA1c in the 2^nd^ trimester compared to the first one, though only the GD group reached statistical significance (Fig 1), most probably due to an increase in the average postprandial glycemia. GD group HbA1c percentage values in the 3^rd^ trimester were lower than those of the 2^nd^ trimester and different from their control groups (p < 0.05), attributable to a decrease in the life span of the erythrocytes, and a decrease in the average and preprandial glycemia.

### Oxidative stress in healthy pregnant women

MDA concentration and GPx activity in the control group herein did not show variation during pregnancy. Both parameters develop in a parallel way (Fig 2 and Fig 3). A majority of the studies on lipid peroxidation that compare healthy pregnant with non-pregnant women found higher levels in the pregnant group [32–34], with ratios between 1.08 and 3.04, with no relationship between the marker and the amount of the increase. Obviously a normal pregnancy is a condition with higher oxidative aggressions, being the placenta one of its sources [11, 35], post-delivery studies show a decrease of the markers already in the first days following delivery [36].

Longitudinal studies allow us to study oxidative stress along pregnancy. Some authors described progressive increases during pregnancy with significant differences in the second and/or third trimester with respect to the beginning of pregnancy [33, 36–39], other authors did not show any difference during the whole period of pregnancy [40–42].

In the present study GPx activity in healthy pregnant women remain stable during the three trimesters, showing a small non-significant decrease in the second trimester (Fig 3). The activity of the antioxidant enzymes (GPx and SOD in plasma and erythrocytes) and other related substances (selenium and glutathione) are normally diminished with respect to the non-pregnant women [16, 33, 34]. Towards the end of the pregnancy the values are more diverse among different authors: some showed increases [4, 39, 41], others showed decreases [16, 33], and even others did not show any change with respect to the beginning of pregnancy [2, 37, 38, 43]. The big methodological differences among all these reports make it difficult to compare them. Some suggested that the increase of the antioxidant markers could indicate the effort of the system to balance the oxidative damage, i.e. up-regulation of the globular form of serum GPx activity as a compensatory mechanism to counteract increased steady-state levels of activated species, and eventually the depletion of the existing resources.

### Oxidative stress in GD

MDA plasmatic concentrations in the GD group did not show significant variations during the first two trimesters of pregnancy (Fig 2). GPx plasmatic activity during the first trimester is significantly lower than the other trimesters, remaining constant in the second half of pregnancy (Fig 3). The GD group showed higher values of MDA concentration during the whole pregnancy with significant differences in the first and the second trimesters, i.e. before and during diagnosis, and before therapeutic measures were adopted. GPx activities were always lower with significant differences in the first trimester. The first trimester was characterized by euglycemia in both groups and similar levels of HbA1c percentage. The women who will develop GD show higher MDA concentration and lower GPx activity than the ones in the control group, with significant differences, suggesting oxidative stress involvement in the pathogenesis of GD. We have detected a significant direct correlation between MDA concentrations and percentage of HbA1c (Fig 5) so it would be possible that oxidative stress levels may be correlated with glycemia in this phase.

During the second trimester a GD metabolic distress develops, but no treatment is received at that time. HbA1c levels increases significantly and MDA concentration shows statistically significant difference among groups. Simultaneously GPx activity increases and the differences among groups disappear so, as mentioned above we hypothesize that there would be an increase in GPx expression, and therefore in its activity, as an attempt to compensate for the steady state levels of activated species.

Finally, in the third trimester, when the conventional dietary recommendations are applied, glycemic control improves and percentage of HbA1c levels diminish, MDA levels decreased to control values, and GPx activity remains constant. Inverse correlations between serum MDA concentration and GPx activity are found at GD group in the 2^nd^ and 3^rd^ trimesters (Fig 4B and Fig 4D): higher MDA values are correlated with lower GPx activities probably due to the adverse effects of lipid peroxidation products on GPx activity when all data were taken together [44].

Ours is the only study in which women with GD were monitored during the whole period of pregnancy. Wender-Ozegowska et al. [45] studied pregnant women with type I diabetes and showed that there were no changes in MDA concentrations during pregnancy in the group of mothers with a satisfactory glycemic control. Santra showed higher significant MDA concentrations than the control ones, from gestational week 31, and repeating the analysis each four weeks in the 3^rd^ trimester, but values of glycemia or HbA1c were not studied, and furthermore, patients with preeclampsia were present in both groups [46], making the data interpretation more difficult.

There are different ways to understand the antioxidant capacity in GD, due to the several analytical methods which have been employed: high vitamin E levels have been described [46] as well as low antioxidant capacity [15]. Peuchant described significant lower values in erythrocytic GPx in GD and no significant differences in erythrocytic SOD [17], but other authors showed a decrease in erythrocytic SOD [18].

Lipid peroxidation has been related to alterations of prostaglandin synthesis which could be responsible for the fetal malformations attributed to diabetes. Our study does not include biochemical data of the newborn but we did not observe differences in their weight or in congenital malformations (data not shown), as a consequence of a disturbed metabolic control. Even though percentage of HbA1c is significantly higher in the GD group in the 2^nd^ and 3^rd^ trimester, these levels are within the recommended range.

Peuchant found a positive correlation between HbA1c and free erythrocytic MDA in the whole group of pregnant women with diabetes (type I diabetes women plus women with GD) at the beginning of the third trimester [17], but not in each group by itself. In our study when we evaluated the GD and the control groups together, a direct correlation between MDA concentration and percentage of HbA1c was found (Fig 5) and indirect correlations between GPx activity and HbA1c percentage (Fig 6), and between GPx activity and MDA concentration (Fig 4) as well, suggesting certain degree of association between pregnancy and oxidative stress.

Herein we can consider the possibility of predicting GD with the oxidative stress markers evaluated. An early GD diagnosis would be necessary to minimize the fetal exposure to suboptimal metabolic conditions and prevent perinatal complications. Our data on oxidative stress show significant differences among groups at the beginning of pregnancy, i.e. before GD diagnosis. Further and larger series would be necessary to confirm the predictive value of this assay.

## Limitations of the study

808 patients were screened during 1^st^ trimester of pregnancy and only 36 patients per group were studied. Although this is certainly a weakness of this study, the data herein represent one of the largest series studied for oxidative stress markers in gestational diabetes. To ascertain that the usual vitamin supplements recommended play a role in the amelioration of oxidative stress markers, the blood levels of those vitamins could have been mentioned. However, since both groups were offered the same protocol (exercise, diet and vitamin supplements) this bias if any, applies to both groups.
